# Occupational Diseases in Art Conservators and Restorers: A Systematic Review

**DOI:** 10.3390/healthcare13070819

**Published:** 2025-04-04

**Authors:** Maria R. Ferreira, André V. Brito, Ricardo J. Fernandes

**Affiliations:** 1 Faculty of Engineering, University of Porto, 4200-465 Porto, Portugal; up202312042@edu.fe.up.pt; 2 Centre of Research, Education, Innovation and Intervention in Sport (CIFI2D), Porto Biomechanics Laboratory (LABIOMEP), Faculty of Sport, University of Porto, 4200-450 Porto, Portugal; up201902341@fade.up.pt

**Keywords:** art, cultural, conservator, restorer, disease, illness, infection, sickness

## Abstract

**Background/Objectives**: Although cultural heritage conservators and restorers face consistent exposure to a multifaceted range of occupational hazards, research on their health remains limited. This systematic review aims to explore and synthesize the prevalence and types of occupational diseases among conservators and restorers of cultural heritage. It also intends to map populations, interventions, contexts and other relevant information to assess the current state of knowledge and identify gaps in the literature on the occupational health of conservation and restoration professionals. **Methods**: The systematic review followed PRISMA 2020 guidelines and the Cochrane handbook. Eligible studies were identified through comprehensive searches of databases, and inclusion criteria were applied to select relevant articles. The protocol was designed according to PRISMA 2020, Prisma-ScR guidelines and the Cochrane handbook. The searches were conducted on 23 May 2024 in PubMed, Scopus and Web of Science (core collection). The risk-of-bias assessment was performed using the Cochrane method for non-randomized studies (RoBANS). **Results**: Respiratory symptoms were the most prevalent occupational health issue, affecting 28% of cases. General symptoms and abdominal issues each accounted for 20% and 18%, respectively, while musculoskeletal disorders were reported in 14% of cases, primarily affecting the neck, back, shoulders and wrists due to prolonged static postures and repetitive movements. Dermatological and irritation manifestations were reported in 10% of cases. Additionally, 10% of cases involved specific diseases such as pneumonia and cancer. The risk-of-bias assessment revealed significant methodological heterogeneity, with notable gaps in exposure assessment and disease outcome reporting across studies. **Conclusions**: This analysis highlights the different health risks faced by conservators and restorers of cultural heritage, underscoring the need for standardized methodologies and prospective studies to increase the data on occupational risks.

## 1. Introduction

Cultural heritage treasures are community assets that reflect human legacy and its cultural values, enhancing community solidarity and social integration [1]. Conservation includes actions to safeguard heritage and ensure its accessibility for present and future generations. This involves preventive preservation, reconstruction and restoration [1] from paintings and sculptures to historical documents and archaeological artifacts [2]. Historically, conservation and restoration were carried out without scientific methods and often disregarded long-term outcomes. Restoring art works was common, but there was little understanding of materials, and the techniques were based on trial and error. The field of conservation emerged especially during the 19th and early 20th centuries, characterized by the development of standardized methodologies and the incorporation of scientific and technological methods for material analysis and restoration. The conservation–restoration area remains inadequately defined, and workers are universally designated as conservators or restorers, regardless of their qualifications or specialization [3].

Professional ethics and artifact treatment have given efforts to define this area, distinguish it from others and also provide specific qualifications. Conservators and restorers work independently in museums, official heritage protection services or private conservation [2]. Conservation–restoration training is primarily offered at universities and cultural institutions, in which students develop knowledge of historical foundations, modern techniques and safety protocols, including the use of chemical products. Workshops and laboratories provide essential practical experience, allowing experienced professionals to share traditional methodologies and skills with beginners. Similarly, conferences and professional courses are essential for sharing knowledge, interdisciplinary collaboration and improving expertise [3]. These professionals are exposed to unsafe working conditions that can affect their health and safety, causing physical or psychological injuries [4]. The common risks are classified as biological (e.g., fungi and bacteria), physical (e.g., thermal environments and radiation), ergonomic (e.g., repetitive motions and poor posture), mechanical (e.g., impacts and collisions), psychosocial (e.g., intense work rhythms and monotony) and chemical (e.g., exposure to volatile organic compounds like solvents and varnishes) [5].

Studies on the indoor air quality in museums show that it directly affects health, influenced by pollutants, the internal microclimate and exposure time. High temperatures can lead to physical and mental exhaustion, while low temperatures cause chills and vascular constriction. In addition, high humidity fosters mold, increasing allergies and asthma, while low humidity leads to nasal dryness, skin irritation and allergies [6]. In conservation laboratories, even without water damage, dangerous microorganisms like Aspergillus fumigatus and Staphylococcus have been detected [6]. Studies conducted in two restoration, conservation and archival studios of cultural heritage institutions also identified exposure to chemicals such as solvents (e.g., ethanol, acetone, benzene and xylene) and compounds like turpentine, ammonia and epoxy resins, with risks varying according to specific restoration activities [7].

Similar occupational risks are observed in laboratory workers, who are frequently exposed to hazardous chemicals and inadequate safety measures. Research highlights that laboratory environments often lack sufficient compliance with regulatory safety standards, leading to prolonged exposure to chemical agents such as formaldehyde and xylene, which are known to cause respiratory issues, skin irritation and neurological symptoms. Additionally, deficiencies in proper ventilation systems and personal protective equipment usage have been identified as contributing factors to increased occupational health risks in these environments [8]. The Occupational Safety and Health Administration (OSHA) has established laboratory safety standards, yet many facilities fail to implement these measures effectively, resulting in preventable workplace hazards. By drawing parallels between conservation professionals and laboratory workers, it becomes evident that improvements in safety protocols, exposure monitoring and regulatory enforcement are essential to mitigating health risks in both fields [8].

Additionally, studies have highlighted work-related musculoskeletal disorders (WMSDs) as a major health concern among conservators, particularly affecting those working in textile conservation. Prolonged static postures, repetitive movements and awkward positions contribute to musculoskeletal issues, including neck, back, shoulder and wrist pain, which can lead to chronic conditions if not properly managed [4,9]. These ergonomic risks are often overlooked, despite their significant impact on worker well-being. Two scoping reviews have already been published focusing on the occupational risks of conservator-restorers, associated with exposure to fungi [6] and work-related diseases [10].

To mitigate the occupational hazards faced by conservators and restorers, preventive measures have been categorized into four areas: preventive maintenance, protective equipment, ergonomic improvements and health promotion. Maintenance strategies include high-efficiency exhaust systems, pollutant control and laser equipment upkeep, alongside building maintenance to prevent water infiltration and biological contamination [6,11]. Personal protective equipment (PPE) such as gloves, gowns and respirators is widely recommended, though proper training is essential for effectiveness. Restricted areas for laser use serve as collective protective measures [11,12]. To address work-related musculoskeletal disorders (WMSDs), institutions have introduced ergonomic chairs with footrests, Pilates sessions and workplace ergonomics workshops, along with frequent breaks and medical supervision [4,9]. These initiatives reflect a growing awareness of occupational health risks in conservation, reinforcing the need for integrated safety protocols in daily practice.

However, few studies analyze illnesses and how they are manifested by workers in real work contexts, which should motivate researchers to explore this topic. The aim of this systematic review was to explore and synthesize the available data on the prevalence and types of work-related diseases among art conservators and restorers. It also maps populations, interventions, contexts and other relevant information to explore the state of the existing knowledge and highlight gaps in the literature.

## 2. Materials and Methods

### 2.1. Eligibility Criteria

The systematic review protocol was registered on the Open Science Framework platform under the registration number (https://doi.org/10.17605/OSF.IO/WUJBK), following the PRISMA 2020 [13], Prisma-ScR guidelines [14] and the Cochrane handbook [15]. Original articles were eligible for inclusion if they were published in journals, in the English language, and there were no restrictions on the publication date. The research topics were as follows: (i) participants: conservation, restoration professionals and cultural heritage workers; (ii) types of work-related diseases seen in this population; (iii) symptoms most frequently reported by these professionals; (iv) diagnostic methods used to identify these diseases.

### 2.2. Information Sources

The searches were carried out on 23 May 2024 in PubMed, Scopus and Web of Science (main collection), applying the filters of document type (articles only) and language (English). After automated and manual searches, the reference lists and relevant reviews of the included studies were examined. Using the snowballing technique, no articles relevant to this study were obtained.

### 2.3. Search Strategy

The Boolean operators AND/OR were used, and each database’s full search strategy is available in the Supplementary Materials. The general search strategy used free-text terms: in the title and abstract only (art OR cultural) AND in all fields (conservator OR restorer) AND (disease OR illness OR infection OR sickness).

### 2.4. Selection Process

M.R.F. and A.V.B. independently examined all the records in the database manually by reading the title and abstract, as well as screening for snowballing citations, and disagreements were resolved by R.J.F.

### 2.5. Data Collection Process and Data Items

M.F. and A.B. independently collected the data, and no automation tools were used. The data items included the workplace characterization (e.g., the studio or laboratory), quantification methodologies (e.g., symptoms, diseases), additional data (e.g., participant characteristics) and protocol information (e.g., questionnaires, medical examinations).

### 2.6. Study of Risk of Bias

This evaluation utilized Cochrane’s risk-of-bias tool for non-randomized studies (RoBANS), examining six specific domains: (i) selection of participants, addressing issues such as inadequate participant inclusion; (ii) confounding variables, focusing on inadequate confirmation and consideration of these variables; (iii) exposure measurement, highlighting cases with insufficient descriptive information; (iv) blinding of outcome assessments, considering the potential impact of prior knowledge of interventions; (v) incomplete outcome data, noting instances of limited available information and (vi) selective outcome reporting, identifying issues such as unregistered protocols and the reporting of results from analyses not specified in the methods.

## 3. Results

### 3.1. Study Selection

**Figure 1:**
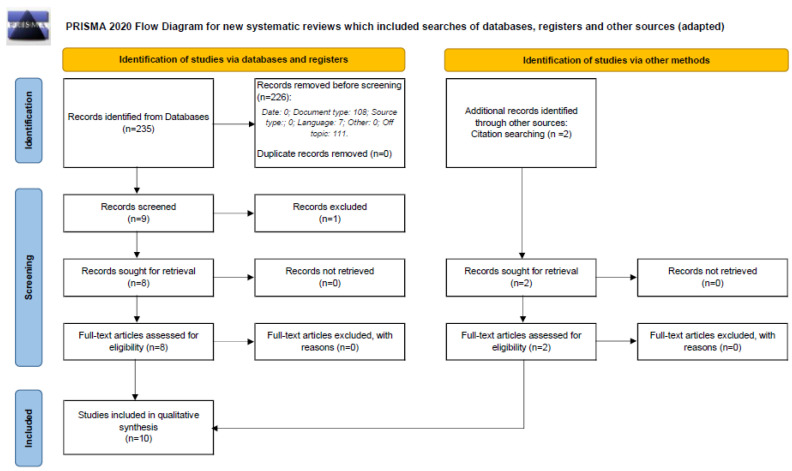
Reporting items for systematic reviews and meta-analyses (PRISMA) 2020 flow diagram.

Figure 1 shows a flowchart diagram of the study selection process. The initial database search yielded 235 records, of which, 108 were not journal articles; 7 were not in English and 111 were excluded based on title and abstract review. Nine full texts were analyzed for eligibility, and one was removed because the population was not eligible (fungi and bacteria). Two further studies were identified through snowballing citation tracking and were included in the synthesis, resulting in a total of ten studies.

### 3.2. Study Characteristics

#### 3.2.1. Publication Year and Research Topics

Table 1 presents the main conclusions of each included study, with specific references provided in the text where necessary. The available literature on work-related diseases has increased in recent years, reflecting a growing interest in research on workers’ health and exposure to adverse working conditions.

**Figure 2:**
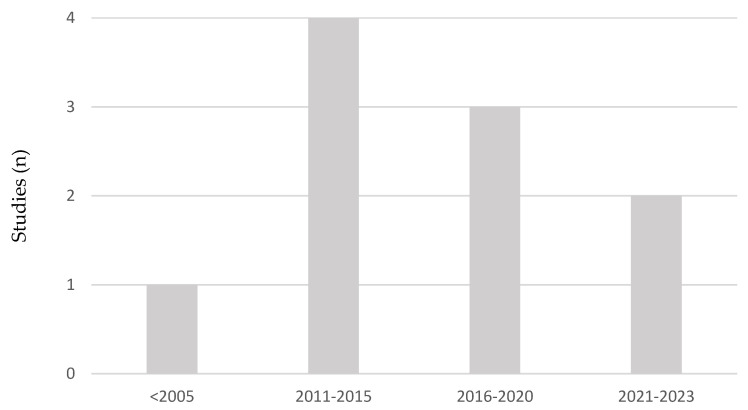
Shows the distribution of studies on work-related diseases in the conservation–restoration sector over time. The first study dates back to 1982, with no publications identified between 2005 and 2010. Research peaked between 2011 and 2015, followed by a decline in recent years.

The analyzed studies span publications from 1982 to 2023. As shown in Figure 2, one article published in 1982 [16] was identified, representing the only record on work-related diseases in the conservation–restoration sector available from the 20th century. Despite its age, this article was considered relevant for addressing pioneering aspects of professionals’ health in this field.

The temporal distribution of the studies revealed that no articles were published between 2005 and 2010, which may indicate a gap in scientific production on the subject during this period. However, starting in 2011, there was an increase in the number of publications, with a total of four articles identified between 2011 and 2015 [7,9,12,17]. This number, although significant, decreased in the following years, with three articles published between 2016 and 2020 [10,18,19] and only two in the most recent period (2021–2023) [4,20]. These findings suggest a peak of interest in the subject between 2011 and 2015, followed by a gradual decline in the volume of publications in recent years.

The evidence on work-related diseases in these studies was extracted from the results of medical examinations and worker questionnaires. Most of the analyzed studies used mixed methods, combining clinical assessments with self-reported symptoms to identify occupational diseases. However, two studies relied exclusively on self-reported symptoms from workers, without medical examinations for confirmation.

The analysis of these data indicates that, although there has been growth in the literature on the subject in recent years, the decline in the number of recent studies may suggest reduced attention to the topic or a possible stabilization of academic interest in the field. These findings highlight the need for ongoing research to further understand the impact of working conditions on the health of exposed professionals.

#### 3.2.2. Participants Characteristics

A total of ten relevant articles were analyzed, nine of which adequately described the study population and provided detailed information about the participants [16,17,18,19,20]. One article [10] did not provide specific information about the study population, and three presented case studies that involved only individuals [12,16,18]. An analysis of the nine articles describing the study population revealed that 45% of the participants were male. The age of the study population ranged from a minimum of 27 years to a maximum of 65 years [19]. In terms of exposure to working conditions, the studies examined show a considerable range, from 3 weeks [12] to 40 years [4,7,17]. However, there are studies that lack information on the age of the population and the years of occupational exposure [10,20].

### 3.3. Environmental Conditions and Experimental Protocol

The workplaces in which occupational exposures occurred included museums [19,20], conservation laboratories with ventilation and poorly ventilated studios [12] and textile conservation workshops [4,9]. The conditions of the workplaces were not mentioned in two articles studies [12,18]. The experimental protocol of the selected articles includes medical interviews [7,17], medical examinations [4,7,16,17,18,19], physical examinations [7,17,18], questionnaires [7,9,19,20], the administration of medication [12,16,18], ergonomic assessments such as the Quick Exposure Check (QEC) [4,9] and the assessment of indoor air quality in the workplaces compared to outdoor air [10].

### 3.4. Outcomes

**Figure 3:**
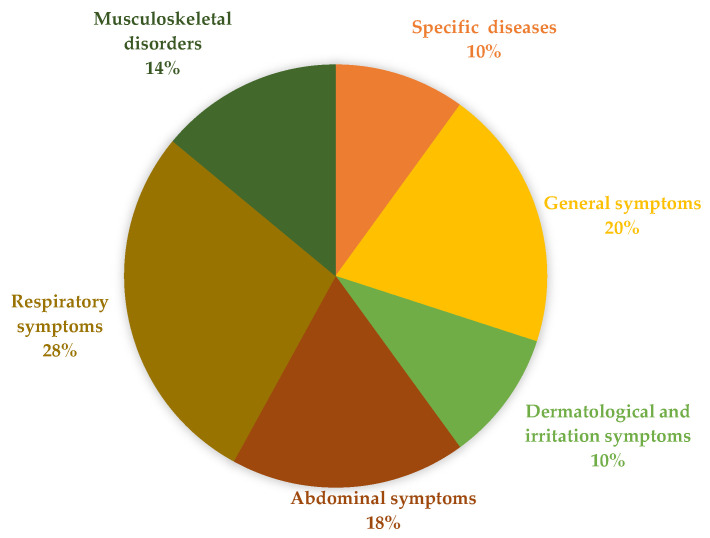
The bar chart illustrates the distribution of symptoms and diseases associated with occupational exposure among textile conservators. Respiratory symptoms (28%) were the most prevalent, including dry cough, nasal congestion, throat irritation, hypoxemia and potential allergic inflammation. General symptoms (20%) encompassed fatigue, headaches, dizziness, difficulty concentrating and muscle pain. Musculoskeletal disorders (14%) were commonly reported, particularly affecting the neck, back, shoulders and wrists due to prolonged static postures and repetitive movements. Abdominal symptoms (18%) included epigastric discomfort, constipation and abnormal liver enzymes. Dermatologic symptoms and irritations (10%) involved eye and skin irritation, dryness and unpleasant odors. Lastly, specific diseases (10%) included pneumonia, various types of cancer (bladder, lung, liver, kidney and skin) and lead poisoning. The chart visually emphasizes the diverse health risks associated with occupational exposure in textile conservation.

To better understand the symptoms and diseases associated with occupational exposure in the workers studied, a diagram (Figure 3) was created to categorize and quantify the different types of manifestations observed in the articles studied. The categories included general symptoms, respiratory symptoms, abdominal symptoms, dermatologic symptoms and irritations, musculoskeletal disorders and specific diseases. Respiratory symptoms were the most common, accounting for 28% of reported cases. These symptoms include dry cough, rhinorrhea, eye and throat irritation, nasal congestion, nasal hyperreactivity to histamine, worsening of lung function, crackles in the lung bases, hypoxemia, possible allergic inflammation and the possible entry of microorganisms into the respiratory tract [7,10,16,17,19,20], indicating a strong correlation with exposure to solvents such as benzene, toluene and xylene, as well as compounds like ammonia and epoxy resins [6,10].

These findings illustrate the considerable strain on the respiratory tract caused by occupational exposure. General symptoms accounted for 20% of reported cases and included fatigue, headache, dizziness, difficulty concentrating, asthenia, myalgia, irritability, decreased libido and sexual impotence [10,12,16,18,20], often linked to prolonged exposure to volatile hydrocarbons. Musculoskeletal symptoms, a category strongly associated with textile conservators, were also highly prevalent, including neck pain, lower back pain, shoulder pain and wrist discomfort due to prolonged static postures and repetitive movements [4,9]. Abdominal symptoms, which also accounted for 18%, included diffuse muscle pain in the upper and lower extremities, intermittent burning pain in the left upper quadrant of the abdomen, epigastric discomfort, abdominal pain, constipation, pseudo-obstruction, hemolytic anemia and abnormal liver enzymes, were associated with heavy metal poisoning, such as lead and arsenic exposure, leading to persistent abdominal pain, gastrointestinal disorders and altered liver enzyme levels [16,18,19].

Dermatologic symptoms and irritation accounted for 10% of the reported cases and included eye and skin irritation, unpleasant odors, dry air and dampness [10,12,20] and were associated with direct contact with fungicides and solvents. Finally, specific illnesses, which also accounted for 10%, included pneumonia [12], cancers (e.g., bladder, lung, liver, kidney and skin) [19] and lead poisoning [16], reinforcing the need for continuous occupational exposure monitoring. Additionally, the analyzed case studies revealed common patterns and differences in the impacts of chemical and microbiological exposure. For instance, while workers exposed to benzene predominantly reported neurological and respiratory symptoms [7], those exposed to gold dust developed intraalveolar pneumonia [12]. These variations highlight the importance of individualized assessments and the development of specific guidelines for mitigating occupational risks.

### 3.5. Risk-of-Bias Assessment

**Figure 4:**
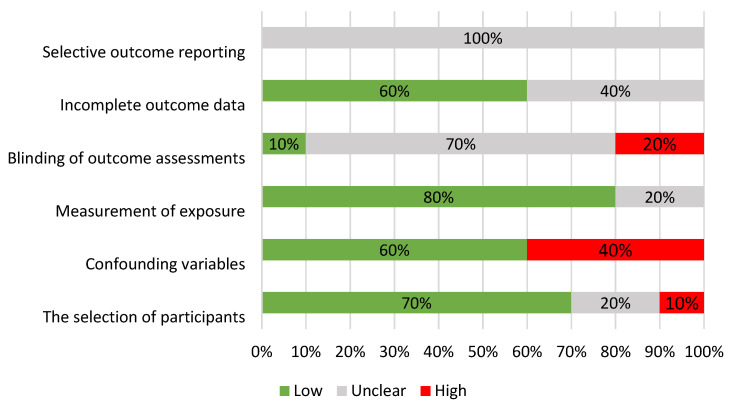
Percentage distribution of risk of bias in non-randomized studies (RoBANS) with low (green), unclear (gray) and high (red) risk [15].

Figure 4 shows the percentage distribution of the risk of bias among the included studies. A high risk was found in the areas of participant selection (10%) [10] and confounding variables (40%) due to missing information on the characteristics of the occupationally exposed participants [10,20]. The assessed risk was unclear in the blinding of outcome assessment (70%) and incomplete outcome data (40%) due to insufficient information. Selective outcome reporting had an unclear risk in all studies (100%) [7,16,20]. Low risk prevailed in most areas, with 60% of the studies showing low risk in incomplete outcome data, 70% in participant selection, 60% in confounding variables and 80% in the measurement of exposure (use of reliable medical examinations). Only 10% of the studies demonstrated a low risk in the blinding of outcome assessments [4,9]. These findings highlight gaps in the methodological quality, particularly in blinding procedures and in reporting completeness.

## 4. Discussion

This systematic review highlights the significant occupational health risks faced by cultural heritage conservators and restorers. The findings emphasize the high prevalence of respiratory symptoms, musculoskeletal disorders and chemical-related illnesses, underscoring the need for standardized methodologies and longitudinal studies to assess long-term health outcomes. The heterogeneity of the included studies, particularly in exposure assessments and diagnostic criteria, represents a major limitation, making it difficult to compare results across different studies and limiting the ability to establish causal relationships between occupational exposures and health outcomes.

### 4.1. Comparative Analysis with Similar Professions

When comparing conservators with workers in similar professions, such as chemical laboratory technicians and museum staff, several common risk factors emerge. Chemical laboratory workers, for instance, face frequent exposure to volatile organic compounds (VOCs) such as formaldehyde and xylene, which have been linked to respiratory issues, skin irritation and neurological symptoms—similar to those reported among conservators [7]. However, unlike laboratory technicians, conservators often work in spaces with poor ventilation, where exposure to solvents and airborne particles is less controlled. Museum employees, on the other hand, are frequently exposed to bioaerosols and fungal spores due to storage conditions and conservation environments, which may contribute to respiratory allergies and infections [10]. This comparative analysis underscores the need for tailored occupational safety measures that address the unique risks faced by conservators, combining aspects of both chemical exposure control (as in laboratories) and biohazard management (as in museum settings).

### 4.2. Preventive Measures

Given the diverse risks identified, the preventive strategies should be multifaceted. Ventilation systems play a critical role in mitigating chemical exposure, particularly in conservation studios in which solvent use is common. High-efficiency particulate air (HEPA) filters, local exhaust ventilation and fume hoods should be implemented to improve air quality and minimize inhalation risks. Studies have shown that inadequate ventilation contributes to increased respiratory issues among conservators [6], highlighting the urgent need for improved air circulation measures.

The use of personal protective equipment (PPE) remains another essential preventive approach. Gloves, masks with organic vapor cartridges and protective clothing can significantly reduce exposure to hazardous substances. However, compliance with PPE use remains inconsistent among conservators due to discomfort and lack of training on proper application [11]. Regular training sessions and improved ergonomic designs of PPE could enhance adherence and effectiveness.

Ergonomic practices are particularly relevant for conservators, who often work in physically demanding postures. Textile conservators, for example, experience a high prevalence of musculoskeletal disorders due to prolonged static postures and repetitive movements [4,9]. Implementing adjustable workstations, ergonomic seating and frequent movement breaks has been shown to reduce strain and prevent chronic pain conditions. Institutions should also consider integrating workplace exercise programs, such as stretching routines or Pilates sessions, which have demonstrated positive outcomes in reducing musculoskeletal discomfort among similar professional groups [4].

### 4.3. Policy Interventions

Beyond workplace-level interventions, policy-level strategies could greatly enhance the occupational health of conservators. Mandatory safety training programs should be introduced to ensure that conservators are fully aware of the risks associated with chemical exposure, poor air quality and ergonomic hazards. Training on proper solvent handling, emergency response to toxic exposures and the safe use of PPE should be standardized and periodically updated.

Another crucial policy intervention is regular health surveillance for conservators, similar to the occupational health monitoring required for chemical laboratory technicians. Periodic medical check-ups focusing on respiratory function, neurological assessments and musculoskeletal health could facilitate the early detection of occupational diseases, allowing for timely intervention and the prevention of long-term health consequences [8].

Additionally, regulatory frameworks should mandate risk assessments and exposure monitoring in conservation workplaces. Institutions employing conservators should be required to conduct periodic air quality tests, monitor chemical exposure levels and ensure compliance with workplace safety standards. Adopting policies modeled after those used in industrial hygiene for laboratory environments could significantly reduce health risks in the conservation sector.

## 5. Conclusions

This systematic review highlights the significant occupational health challenges faced by cultural heritage conservators and restorers, emphasizing the prevalence of respiratory, general, abdominal, dermatological and musculoskeletal disorders in this profession. These findings underscore the multiple health impacts associated with their working environment and the need for comprehensive interventions. Despite the limitations resulting from methodological heterogeneity and data quality, this study reinforces the urgency of standardized methods, prospective studies and targeted measures to effectively reduce occupational risks.

Collaboration between health professionals, conservation experts and policymakers is crucial to developing and implementing robust safety protocols, improving epidemiological surveillance and protecting the health and well-being of workers in this field. Future research should focus on longitudinal studies with objective health assessments, explore innovative prevention strategies and bridge existing knowledge gaps in the epidemiology of occupational health related to heritage conservation.

Given the clear risks identified, museum administrations, policymakers and cultural heritage institutions must take immediate action to prioritize workplace safety in conservation environments. This includes enforcing stricter regulations on air quality control, ensuring access to personal protective equipment (PPE), investing in ergonomic improvements and mandating regular occupational health monitoring for conservators. By committing to these initiatives, stakeholders can safeguard both the health of conservators and the preservation of cultural heritage for future generations.

## Figures and Tables

**Table 1:** Characteristics of the included trials (authors, conflict of interest/funding, participants, environmental conditions, experimental protocol and outcomes).

(Year) [Reference]	Conflict of Interest and Funding	Participants’ Characteristics (Age and Years of Exposure)	Environmental Conditions	Experimental Protocol	Risk Factors	Key Outcomes (Symptoms and Diseases)	Preventive Measures
Fischbein, A.; Wallace, J.; Anderson, K.E.; et al. (1982) [16]	Funded by National Institute of Environmental Health Sciences Center	43 years old (female) Exposure—six weeks Art conservator	Studio Poorly ventilated room New York City	Routine gynecologic examination, physical examination, Hospitalized for chelation therapy with disodium calcium edetic acid	Exposure to lead	Fatigue, dizziness, diffuse muscle pain in upper and lower extremities, intermittent burning pain in the left upper quadrant of the abdomen, epigastric discomfort and crampy abdominal pain unrelated to eating Elevated blood lead levels (up to 130 mg/dL)	Reevaluation of safety standards; Mandatory documentation of chemical content; Risk assessment for historical materials; Improving ventilation in workspaces.
Varnai, V.M.; Macan, J.; Prester, L; et al. (2011) [7]	Funded by Ministry of Science and Technology of the Republic of Croatia	147 recruited controls (29 men) Age in years, mean (38.6) Exposure—six, five years 56 recruited restorers (22 men) Age in years, mean (37.6) Exposure—6 years	Two Croatian institutions Control group: insurance company and librarians Restorers: in restoration and conservation laboratory	Medical interview, physical examination and medical examinations (venous blood sampling for total IgE measurement, skin prick testing to common inhalational allergens, non-specific bronchial challenge test) Simple questionnaire: medical history (smoking habit, years of working exposure, exposure to dust and chemicals at the workplace and data on ever experienced respiratory symptoms compatible with diagnoses of rhinitis and asthma	Exposure to volatile solvents hydrocarbons, ketones, esters and alcohols	More than two times higher prevalence of nasal hyperresponsiveness to histamine was found in restorers and conservators of cultural heritage compared to control subjects; no deterioration of lung function	Not mentioned
Ribeiro, Pa.A; Girão, F.; Henriques, P; et al. (2011) [12]	Not mentioned	47 years old (man) Exposure—three weeks Restorer of religious art	Not mentioned During three weeks	Imaging studies, lung function tests, bronchoalveolar lavage, lung biopsy, treatment with Prednisolone 40 mg, follow-up computed tomography scan	Exposure to golden dust	Asthenia, myalgia, dry cough, fever, crackles in lung bases, hypoxemia and elevation of inflammatory markers, pneumonia	Elimination of exposure to the causative agent; Use of chemical protective masks; Early diagnosis and etiological investigation; Ongoing health monitoring for workers; Appropriate therapy in case of illness.
Kanceljak-Macan, B.; Trošíc, I.; Varnai, V. M.; et al. (2012) [17]	Funded by medical research and Occupational Health, Zagreb	22 restorers (10 men) Exposure—five years 48 control workers (27 men) Exposure—six years	Two Croatian institutions Control group: without lower respiratory symptoms and with normal ventilatory parameters	Medical interview, physical examination, spirometry, skin prick testing to inhalator allergens	Chemical exposure varied according to restoration/conservation activity, but all the subjects reported being exposed to mixture of organic solvents, including 95% ethanol, acetone, benzine, white spirit, toluene and xylene, and the majority of them were also exposed to turpentine, ammonia, perchlorethylene, fungicides, vinyl polymers and epoxy resins	Restorers: Male: a higher percentage of neutrophils (34 vs. 15.5%), Female: lower proportion of neutrophils in sputum	Not mentioned
Langford, M., Beaumont, M. S., & Annett, D. (2013) [9]	Not mentioned	Textile conservators	Historic Royal Palaces (HRP) collections in the UK Average of 15 years of experience	Observation of work postures, analysis of accident and absenteeism records, ergonomic questionnaire and ergonomics workshop	1. Manually handle loads (moving rolled tapestries to and from the store weighing around 100 kg); Chairs without footrests.	Musculoskeletal disorders (MSDs), lower back pain, neck pain, upper limb pain due to static postures and repetitive movements	Adjusting working environments by creating customized equipment to carry out tasks in new ways; Ergonomics workshops and Pilates classes; Formalization of work procedures; Investment in additional fixed and mobile lighting.
Górny, R. L.; Harkawy, A. S.; Lawniczek-Walczyk, A.; et al. (2016) [10]	Not mentioned	Workers not defined	Nine naturally ventilated conservation laboratories with no history of water damage	Viable bioaerosol stationary samples were collected in both outdoor and indoor environments using 6-stage Andersen impactor Stationary and personal indoor bioaerosol measurements were carried out using both Gesamtstaubprobenahme an der Person and Button filter samplers, simple questionnaire	Exposure to microbiological agents	Headaches, fatigue, eye and throat irritation, as well as rhinorrhea and allergic inflammation	Precise assessment of workers’ contamination level; Mechanical isolation of the working environment; Use of glove boxes; Installation of local exhaust ventilation benches; Control of microclimate parameters, especially relative humidity.
Costa-Moreira, P.; Coelho, R.; Pita, I.; et al. (2019) [18]	Not mentioned	39 years old (male) Exposure—five months Art conservator	In an early 20th-century church	Physical examination, the blood tests, abdominal radiographs, tomography, a colonic pseudo-obstruction, an upper endoscopy, a colonoscopy and a liver biopsy, chelation with calcium disodium edetate was started at a dose of 20 mg/kg/d	Exposure to lead	Abdominal pain, constipation, irritability, concentration difficulties, decreased libido and sexual impotence, pseudo-obstruction, hemolytic anemia and abnormal liver chemistries	Not mentioned
Deering, K.; Spegel, E.; Quaisser, C.; et al. (2020) [19]	Funded by the Deutsche Bundesstiftung Umwelt DBU	28 museum employees (20 men) between 27 and 65 years of age Exposure—six months, worked at least ten hours per week	In Museum für Naturkunde Berlin	Two blood samples and five urine samples were taken from each participant (during a working week), a questionnaire (work activity, exposure and information on fish and seafood intakes)	Exposure to arsenic, mercury and organochlorine pesticides	Cancer such as bladder, lung, liver, kidney, skin cancer, affect the development processes of infants during the prenatal and early postnatal period, the nervous, respiratory, immune, cardiovascular and endocrine system with various health issues	No collection objects should be stored beside permanent workplaces in order to avoid a hazardous substance transfer, and long object transport routes within the building should be shortened; The contamination in the air can be reduced by frequent cleaning intervals and high air exchange rates.
Pinheiro, A. C., & Ramos, A. (2021) [4]	Funded by the Foundation for Science and Technology	6 textile conservators in Portugal, aged 39–62 years, experience ranging from 12 to 40 years	Textile conservation workshops; long hours in static postures; strenuous tasks such as textile consolidation	Nordic Musculoskeletal Questionnaire, Quick Exposure Check (QEC) to assess ergonomic risk	1. Incorrect postures on the tapestry looms; Workers used wooden chair made specifically for the textile department in the 1980s–1990s.	Neck pain, back, shoulder and hand/wrist pain	Taking frequent breaks; Performing specific exercises to release the tension accumulated in those areas most affected (e.g., lower back); Purchase of chair with footrest.
Ilies, D.C.; Herman, G.V.; Safarov, B. et al. (2023) [20]	Funded by the Deanship of Scientific Research	Visitors and employees (250 respondents—108 men)	In Darvas-La Roche Museum House (Romania) Too low temperature, dry, unventilated air, as well as a large amount of dust in suspension	Questionnaire with 11 items (of the indoor environment and experienced symptoms of illness) September 2023–March 2023	The air quality	Disease symptoms (nasal congestion, eye and skin irritations, coughs, migraines, frequent colds, etc.) and/or discomfort sensations (dry air, excess humidity, unpleasant smells, etc.)	Not mentioned

## Supplementary Materials

The following supporting information can be downloaded at: https://www.mdpi.com/article/10.3390/healthcare13070819/s1, File S1. PRISMA—Data organization. Reference [21] is cited within the Supplementary Materials.
